# Paradoxical Effect of LTB_4_ on the Regulation of Stress-Induced Corticosterone Production

**DOI:** 10.3389/fnbeh.2019.00073

**Published:** 2019-04-16

**Authors:** Gisele A. Locachevic, Morgana K. B. Prado, Karina F. Zoccal, Priscilla A. T. Pereira, Carlos A. Sorgi, Mariza Bortolanza, Ana Paula F. Peti, Manoela V. Fogaça, Francisco S. Guimarães, Elaine Del Bel, Lúcia H. Faccioli

**Affiliations:** ^1^Departamento de Análises Clínicas, Toxicológicas e Bromatológicas, Faculdade de Ciências Farmacêuticas de Ribeirão Preto, Universidade de São Paulo, São Paulo, Brazil; ^2^Departamento de Morfologia, Fisiologia e Patologia Básica, Faculdade de Odontologia de Ribeirão Preto, Universidade de São Paulo, São Paulo, Brazil; ^3^Departamento de Farmacologia, Faculdade de Medicina de Ribeirão Preto, Universidade de São Paulo, São Paulo, Brazil

**Keywords:** chronic unpredictable stress, depression, 5-lipoxygenase, prostaglandins, leukotrienes, IL-1β, caspase-1, glucocorticoid receptor

## Abstract

Depression is a mental illness with a complex and multifactorial etiology, which has been associated with stress and inflammation. Infections, autoimmune diseases, envenomation, and trauma induce an inflammatory response that is characterized by increasing levels of circulating cytokines (e.g., IL-1β) and lipid mediators [e.g., PGE_2_ and leukotrienes B_4_ (LTB_4_)]. Recently, we showed that LTB_4_ production by the 5-lipoxygenase (5-LO) pathway regulates IL-1β and PGE_2_ release, reducing tissue damage in a model of sterile inflammation. Since IL-1β and PGE_2_ increase in serum of stressed patients and potentially trigger depression, we used an animal model of chronic unpredictable stress (CUS) to investigate the potential impact of LTB_4_ over depression-like symptoms. At basal conditions, 5-LO deficiency (*Alox5*^−/−^) reduces the preference for sucrose, while inducing a higher immobilization time on the tail suspension test when compared 129*sv*. Moreover, *Alox5*^−/−^ mice present increased caspase-1 expression and elevated levels of IL-1β, IL-17 and PGE_2_ in the spleen, with increasing corticosterone levels in the frontal cortex but reducing systemic levels. Compared to 129*sv* mice, CUS induced higher levels of systemic, frontal cortex and hippocampal corticosterone, and also reduced sucrose preference, increased levels of splenic IL-1β, IL-17 and PGE_2_ and reduced levels of LTB_4_. Interestingly, CUS exposure did not alter the reduced sucrose preference shown by *Alox5*^−/−^ mice but greatly enhanced splenic PGE_2_ production. Compared to *Alox5*^−/−^ mice at basal conditions, CUS exposure also increased levels of systemic corticosterone, which remained lower than those of CUS-129*sv* animals. We also observed that treatment with LTB_4_ decreased caspase-1 expression and systemic levels of corticosterone in CUS-*Alox5*^−/−^ mice but there was no significant impact on the reduced sucrose preference. Our results demonstrate that LTB_4_ controls the hypothalamic-pituitary-adrenal (HPA) axis by regulating levels of systemic corticosterone associated with the repression of caspase-1 expression and production of inflammatory mediators. One limitation of our study is that 129*sv* and *Alox5*^−/−^ mice were not littermates, not sharing, therefore, the same intra-uterine and preweaning environment. Even so, taken together our results indicate that 5-LO activity is critical for the regulation of stress-induced symptoms, suggesting that the *Alox5*^−/−^ mouse could be a natural model of corticosterone-independent reduced reward sensitivity.

## Introduction

Depression is a serious public health problem and one of the most common psychiatric disorders in Western countries. It may lead to suicide, a tragic fatality accounting for mortality of 800,000 people every year (World Health Organization (WHO), 2017). Depressed patients often exhibit symptoms such as sadness, loss of interest or pleasure (anhedonia), feelings of guilt or low self-esteem, disturbances in sleep or appetite, low energy, and a reduced ability to concentrate (Zunszain et al., 2011). An association between stress, immune response, and psychological disorders has been proposed over the last few decades (Guan et al., 2014). A considerable number of evidence demonstrates that multiple aspects of the immune response are altered during stress or depressive episodes (Maes et al., 1997; Tymen et al., 2013). Indeed, the immune response has been shown to contribute to the pathophysiology of these disorders (Irwin and Miller, 2007). In conditions of chronic psychological stress, such as major depressive disorders, both the cellular and humoral immunity can be persistently activated and contribute to depression.

Depressed and chronically stressed individuals exhibit higher levels of circulating proinflammatory cytokines, chemokines, acute phase proteins, and soluble adhesion molecules or even cerebrospinal fluid (Smith, 1991; Raison et al., 2006; Dantzer et al., 2008). Interestingly, systemic or central administration of cytokines such as IL-1β, IL-6 or TNF-α promotes behavioral perturbations in rats and mice (Dantzer, 2001). Furthermore, mononuclear cells isolated from depressed patients exhibit increased NLRP3 inflammasome and caspase-1 activation, which culminates in elevated systemic levels of IL-18 and IL-1β (Alcocer-Gómez et al., 2014).

Recently, Zhang et al. (2015) reported that mice subjected to chronic mild stress (CMS) have increased IL-1β levels, caspase-1 activity, and NLRP3 inflammasome activation in the hippocampus. Pharmacological inhibition of the inflammasome decreased both IL-1β levels and the CMS-induced depressive-like behavior. Stress induced by pathogenic or non-pathogenic (sterile) stressors triggers the NLRP3 inflammasome in myeloid cells, resulting in the activation of caspase-1, which cleaves the precursor forms of IL-18 and IL-1β cytokines, leading to release of the active cytokines (Strowig et al., 2012; Zhang et al., 2015; Miller and Raison, 2016).

Glucocorticoids, such as cortisol (in humans) and corticosterone (in rodents), are anti-inflammatory hormones (Zen et al., 2011) that increase during chronic stress and depression (Pitman et al., 1988; Cohen et al., 2012; Herane Vives et al., 2015). However, pro-inflammatory cytokines induce glucocorticoid-receptor (GR) resistance (Jeon and Kim, 2016; Rodriguez et al., 2016), suggesting an impairment of glucocorticoid-mediated immunoregulatory mechanisms. Glucocorticoid production is regulated by a negative feedback loop involving GR activation (Smith and Vale, 2006). Furthermore, both isoforms of the enzyme 11β-hydroxysteroid dehydrogenase (11βHSD1 and 11βHSD2) regulate glucocorticoid concentrations. The 11βHSD1 isoform converts inactive 11-ketometabolites into active glucocorticoids in the presence of NADPH. In contrast, the 11βHSD2 isoform metabolizes active glucocorticoids to produce inactive 11-ketometabolites using NAD^+^ as a cofactor (Krozowski et al., 1999).

In association with the 5-lipoxygenase-activating protein (FLAP), the enzyme 5-lipoxygenase (5-LO) catabolizes arachidonic acid into leukotrienes (LT) B_4_ (LTB_4_), C_4_ (LTC_4_), D_4_ (LTD_4_) and E_4_ (LTE_4_; Funk, 2001; Harizi et al., 2008; Rådmark et al., 2015). LTs have been shown to regulate the hypothalamic-pituitary-adrenal (HPA) axis (Hirai et al., 1985). Sato et al. (2008) demonstrated that 5-LO metabolites down-regulate 11βHSD2 activity in trophoblasts *in vitro*. Moreover, Uz et al. (1999) have shown that glucocorticoids increase the transcriptional and protein levels of 5-LO in the brain. Interestingly, a polymorphism in the gene encoding the enzyme leukotriene A_4_ hydrolase, which catalyzes LTB_4_ formation (Haeggström and Funk, 2011), has been associated with susceptibility to depression, especially in women (Zhao et al., 2009). Furthermore, the FLAP inhibitor MK886 induces antidepressant-like effects in mice submitted to the forced swimming test (Uz et al., 2008).

Recently, our research group demonstrated that PGE_2_ increases IL-1β production by a cyclic adenosine monophosphate (cAMP)-dependent mechanism in a model of sterile inflammation (Zoccal et al., 2016). Moreover, we identified that LTB_4_ regulates this process by reducing intracellular cAMP levels, which in turn inhibits IL-1β release by innate immune cells (Zoccal et al., 2016). Considering that IL-1β and PGE_2_ are potential triggers of depression (Müller et al., 2006; Goshen and Yirmiya, 2009), and that LTB_4_ is able to repress the production of these mediators (Zoccal et al., 2016), we hypothesized that 5-LO deficiency would increase the susceptibility to depression. To investigate this hypothesis, we submitted *Alox5*^−/−^ (5-LO deficient) and 129*sv* mice to chronic unpredictable stress (CUS), an experimental model that induces depression-like behaviors such as decreased sucrose preference. Compared to 129*sv* mice at basal conditions, *Alox5*^−/−^ animals exhibited an intrinsically lower preference for sucrose and higher levels of IL-1β, IL-17, PGE_2_, adrenocorticotropic hormone (ACTH), and caspase-1 expression. In contrast, these mice present lower 11βHSD2 and GR expression, as well as reduced levels of corticosterone, 11-dehydrocorticosterone (11DHC), and circulating neutrophils. As expected, 129*sv* mice exposed to CUS displayed increased levels of splenic proinflammatory and stress-induced mediators, and signs of reduced sucrose preference, which could be associated with anhedonia (Willner, 1997). Intriguingly, most of the analyzed parameters remained at the same levels before and after CUS exposure in *Alox5*^−/−^ mice. These levels were comparable to those of CUS-129*sv*. Importantly, exogenous LTB_4_ administration to CUS-*Alox5*^−/−^ mice decreased systemic corticosterone levels and caspase-1 expression without affecting sucrose preference.

## Materials and Methods

### Animals

Male mice (7–8-weeks-old) were used for the experiments. Animals lacking the 5-LO gene (*Alox5*^−/−^) were age-matched in all procedures with their genetic background, the wild-type 129*sv* mouse strain (Chen et al., 1994). The *Alox5*^−/−^ (129-*Alox5*^tm1Fun^/J) and 129*sv* mice were obtained from The Jackson Laboratory (Bar Harbor, ME, USA) and were bred in the Faculdade de Ciências Farmacêuticas de Ribeirão Preto, Universidade de São Paulo (Ribeirão Preto, SP, Brazil). C57Bl/6 mice, obtained from the Faculdade de Ciências Farmacêuticas de Ribeirão Preto, Universidade de São Paulo, were used as controls to validate our methodology used in the 129*sv* mice. Mice were kept in 12 h light/dark cycles, with *ad libitum* access to water and food, except for the period of exposure to stressors (described in “Chronic Unpredictable Stress and Sample Collection” section). All procedures were performed according to the ethical standards established by the National Council for Control of Animal Experimentation (CONCEA) and approved by the Ethics Committee on Animal Use (CEUA) of the Campus of Ribeirão Preto (Protocol number: 14.1.290.53.5).

### Chronic Unpredictable Stress and Sample Collection

C57BL/6, 129*sv*, and *Alox5*^−/−^ mice were subjected over a period of 14 days to the following daily stressors, which were applied randomly: an alteration in bedding (sawdust removal and replacement with wet sawdust having approximately 5 mm deep water) for 24 h; reversal of the light/dark cycle; 15 min forced swimming; 1 h restraint in a tube. The last stressor was always water and food deprivation for 20 h. The next day, animals underwent a sucrose preference test to evaluate depressive-like behavior (Willner, 1997). Non-stressed animals from each strain were used as controls and these animals were also subjected to 20 h without food and water before the sucrose test. In some experiments, mice exposed or not to CUS were also treated with specific drugs or vehicle, as described below.

At the end of the experiment, mice were anesthetized (75 mg/kg ketamine e 10 mg/kg xylazine) and the blood was immediately collected by cardiac puncture for the analysis of circulating leukocytes. All blood samples were collected at 12:00 pm and in the presence or absence of an anticoagulant and mice were weighed at day zero, and again at day fifteen. Next, the animals were euthanized by cervical dislocation. The spleen and adrenal glands, and the cerebellum, frontal cortex, and hippocampus were weighed after collection.

### Sucrose Preference Test

129*sv* and *Alox5*^−/−^ mice were individually housed and deprived of food and water (the last stress stimulus) for 20 h. Next, a glass bottle with water and another with water containing 1% sucrose was placed in each cage. The bottles were initially weighed and consumption was monitored after 1 h. For this purpose, the glass bottles were weighed again to estimate the liquid ingestion. The sucrose preference was calculated as described previously (Guo et al., 2009) with the following formula: sucrose consumption/(sucrose consumption + water consumption).

### LTB_4_ Treatment *in vivo*

*Alox5*^−/−^ mice were intraperitoneally (i.p.) injected with LTB_4_ (50 ng in 100 μL; Cayman, Ann Arbor, MI, USA) during the morning. Treatment initiated 1 day before CUS, followed by administration twice a day (12/12 h) during the 14 days of CUS exposure. LTB_4_ was dissolved in ethanol and diluted in aqueous solution containing 0.45% NaCl, 0.25% dextrose, and 0.01% bovine serum albumin (BSA) followed by filter sterilization. The same volume of ethanol was added to the same aqueous solution to be used as the vehicle control. The LTB_4_ dose was selected based on previous studies (Zoccal et al., 2016, 2018).

### Leukocyte Counts and Adrenocorticotropic Hormone (ACTH) Quantification

Blood samples were collected and immediately used for total and differential counts of circulating leukocytes as previously described (Pereira et al., 2013). In one specific set of experiments, blood was collected in tubes with EDTA (BD Microtainer, BD Biosciences, San Jose, CA, USA), centrifuged (400× *g* for 10 min at 4°C), and the remaining plasma was stored at −20°C. ACTH concentrations were determined with a chemiluminescent immunoassay (CLIA) technology (Liaison ACTH, 313221, DiaSorin, Sallugia, Piemonte, Italy) employing a Liaison Analyzer (DiaSorin, Sallugia, Piemonte, Italy), following the manufacturer’s instructions. The sensitivity of the assay was <1,500 pg/mL.

### Mass Spectrometry (MS) Measurements

Corticosterone and 11DHC from serum, frontal cortex, hippocampus, and cerebellum were quantified by mass spectrometry (MS) as previously described (Peti et al., 2018). Tissues were weighed, homogenized (Mixer Homogenizer, Labortechnik, Wasserburg, Bavaria, Germany) in methanol:water (v/v 1:1), centrifuged, and the supernatant was recovered. Supernatants and serum were purified as previously described (Galvão et al., 2016). Samples were analyzed using the mass spectrometer TripleTOF 5600^+^ (Sciex, Foster, CA, USA) coupled with the liquid chromatography system Nexera (Shimadzu Corp., Kyoto, Japan). Data were processed using PeakView and MultiQuant software. For spleens, PGE_2_ and LTB_4_ levels were determined using a LC-MS/MS system Acquity UPLC^®^—Xevo TQ-S (Waters, Milford, MA, USA). Data were analyzed with MassLynx 4.0 and TargetLynx 4.0 softwares.

#### Enzymatic Immunoassay (EIA) Measurements

Corticosterone levels were also determined using a specific enzyme immunoassay kit (Corticosterone ELISA kit, Enzo Life Science, New York, USA) according to the manufacturer’s instructions. The reading was taken at 405–420 wavelength and the results were expressed in ng/mL.

### Quantification of IL-1β and IL-17 by Enzyme-Linked Immunosorbent Assay

Spleens were homogenized (Mixer Homogenizer, Labortechnik, Wasserburg, Germany) and commercially available Enzyme-Linked Immunosorbent Assay (ELISA) kits (R&D Systems, Minneapolis, MN, USA) were used for quantification of IL-17 and IL-1β levels, according to the manufacturer’s instructions. The sensitivity of the assay was <10 pg/mL.

### Determination of T Lymphocyte Populations and Cellular Apoptosis by Flow Cytometry

Spleen cell suspensions were obtained by organ maceration following erythrocytes lysis with ACK lysis buffer (Invitrogen, Carlsbad, CA, USA). Total cell suspensions were adjusted to 1 × 10^6^ cells/mL and flow cytometry was carried out as previously described (Secatto et al., 2012). The following fluorochrome-conjugated monoclonal antibodies were used: anti-CD3 (FITC); anti-CD4 (PE); and anti-CD8 (PerCP; BD Bioscience, San Jose, CA, USA). Samples were analyzed using a FACSCanto Flow Cytometer (BD Bioscience, San Jose, CA, USA). The percentage of CD3^+^CD4^+^ and CD3^+^CD8^+^ double-positive cells were calculated using the FACSDiva software. Apoptosis was evaluated in spleen using an apoptosis detection kit based on annexin V-FITC/propidium iodide (PI) staining (BD Pharmingen, San Jose, CA, USA), according to the manufacturer’s instructions. Briefly, cellular suspensions were washed twice with PBS, centrifuged at 400× *g* for 15 min, suspended in the kit buffer, and then incubated with annexin V in the absence of light for 15 min. PI was added to all samples immediately before data acquisition. A total of 10,000 events were acquired from all samples using the FACSCanto flow cytometer (BD Bioscience, San Jose, CA, USA). The results were evaluated using the FACSDiva software and expressed as the percentage of cells in the initial stage of apoptosis, i.e., positive for annexin V and negative for PI (Locachevic et al., 2015).

### Quantitative Real Time PCR

Adrenal glands and spleens were collected, and total RNA was extracted using PureLink RNA Mini Kit (ThermoFisher Scientific, Waltham, MA, USA), according to the manufacturer’s instructions. RNA concentration and purity were evaluated using a fluorimetric assay (Qubit, Invitrogen, Carlsbad, CA, USA). Complementary DNA (cDNA) was obtained using the High Capacity cDNA Reverse Transcription kit (Applied Biosytems, Foster City, CA, USA), according to the manufacturer’s instructions. Total cDNA was amplified by real-time reverse transcription polymerase chain reaction (RT-qPCR) using TaqMan primers for the *Hsd11b1, Hsd11b2*, and *Casp1* genes employing StepOne Plus (Applied Biosystems, Foster City, CA, USA). *Actb* was used as the endogenous control. The reactions were amplified under the following conditions: denaturation at 95°C for 2 min, followed by 40 cycles of 95°C for 2 min and 60°C for 20 s. The expression of the genes *Hsd11b1* (Mm01251104_m1, Applied Biosystems, Foster City, CA, USA), *Hsd11b2* (Mm00476182_m1, Applied Biosystems, Foster City, CA, USA), and *Casp1* (Mm.PT.58.13005595, IDT, San Diego, CA, USA) were normalized by housekeeping gene expression (*Actb*). The gene expression of 129*sv* mice was considered as the reference (129*sv* mice), being 2^−ΔΔCT^ = 1. The primers were obtained commercially and are proprietary; thus, sequences are not available (TaqMan Gene Expression Assay, Applied Biosystems, Foster City, CA, USA).

### Immunoblotting for Determination of the Glucocorticoid Receptor (GR)

The spleens and hippocampi from mice subjected or not to CUS were isolated and immediately immersed in RIPA buffer supplemented with protease inhibitors (Roche Diagnostics, Basel, Switzerland). After homogenization and centrifugation at 400× *g* for 10 min, the supernatant was used to measure GR expression. Equal amounts of protein (50 μg) from each sample were separated by polyacrylamide gel for electrophoresis (10% Tris-HCl) and subsequently transferred to a nitrocellulose membrane, as previously described (Serezani et al., 2005). The membranes were immunolabeled with an anti-GR monoclonal primary antibody (1:10,000; MCA1390, BioRad Antibodies, CA, USA), or anti-GAPDH antibody (1:5,000; Sigma Aldrich, St. Louis, Missouri, USA) as the endogenous control. Following membranes were incubated with the appropriate horseradish peroxidase (HRP)-conjugated secondary IgG (1:10,000; Cell Signaling, Danvers, MA, USA). ECL reagent (Amersham Biosciences, Little Chalfont, UK) was used to reveal by chemiluminescent reactions using a photo documenter (Alliance 4.7, UVITEC, Cambridge, England, UK). The band densities were determined using the ImageJ program^1^ and normalized by the signal intensity emitted by the endogenous GAPDH control.

### Enzymatic Activity of 11βHSD2

Activity of 11βHSD2 was evaluated as described by Kajantie et al. (2003) and Stewart et al. (1995) in samples of spleen, adrenal gland, frontal cortex and cerebellum from 129*sv* and *Alox5*^−/−^ mice, exposed or not to CUS. Briefly, all samples were homogenized (Mixer Homogenizer, Labortechnik, Wasserburg, Germany) in KCl buffer (0.154 mol/L; pH 7.6) and centrifuged at 1,000 *g* for 10 min 4°C. Protein assay was performed on the supernatant using Coomassie blue staining, as described previously by Medeiros et al. (2008). Supernatants (0.50 g/L of spleen protein, 0.15 g/L of adrenal protein, 0.10 g/L of frontal cortex protein or 0.20 g/L of cerebellum protein) were incubated with a solution containing 1.0 mL of phosphate buffer (0.1 mol/L; pH 7.6), 0.8 nmol/L nicotinamide adenine dinucleotide, and 0.2 mol/L corticosterone in a shaking water bath at 37°C. Spleen proteins were incubated for 30 min and the other tissues for 45 min. Enzymatic reactions were stopped with 1.0 mL of methanol. For extraction, 7 mL of dichloromethane was added to the samples and vortexed for 30 s. The upper phase was removed, 2 mL of water were added, and the tubes were mixed again. The lower phase (organic phase) was collected, dried and resuspended in 40 μL (spleen) or 25 μL (other tissues) of methanol. The enzymatic activity was evaluated by the conversion of corticosterone into 11DHC, which were measured by LC-MS/MS (Peti et al., 2018).

### Statistical Analyses

Data were analyzed using SPSS V. 20 (IBM, USA). After checking for normality and homoscedasticity, the results were analyzed using Student’s *t*-test, two- or one-way analysis of variance (ANOVA), when appropriated. *Post hoc* comparisons were performed with the Newman-Keuls test. Differences were considered significant when *p* < 0.05.

## Results

### *Alox5*^−/−^ Mice Show an Intrinsic HPA Activation and Differential Dynamics of Systemic and Hippocampal Corticosterone

**Figure 1:**
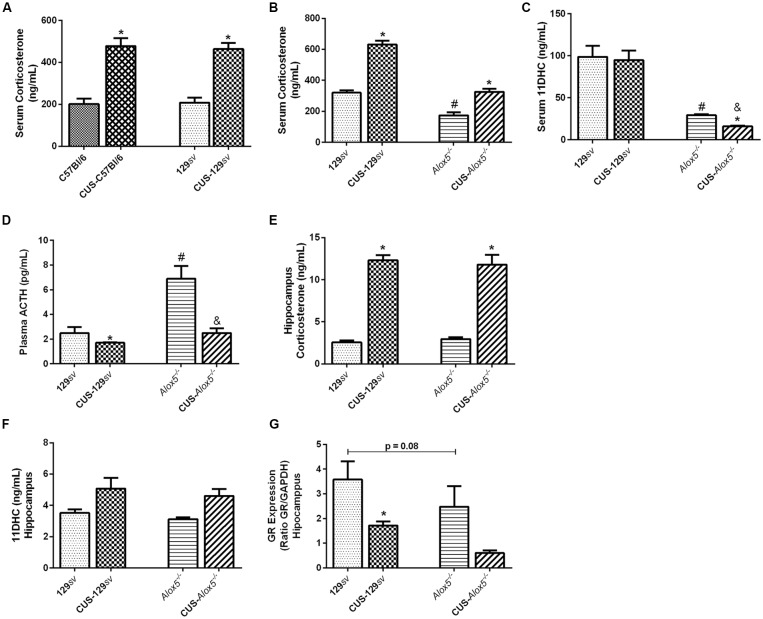
*Alox5*^−/−^ mice exhibit a natural hypothalamic-pituitary-adrenal (HPA) axis activation and produce different corticosterone amounts in the serum and hippocampus. **(A)** The C57Bl/6 and 129*sv* mice were subjected or not to chronic unpredictable stress (CUS) for 14 days. Animals were euthanized on the 15th day and blood was collected for corticosterone quantification. **(B–D)** *Alox5*^−/−^ and 129*sv* mice were subjected or not to CUS for 14 days. Animals were euthanized on the 15th days and blood was collected for quantification of **(B)** corticosterone, **(C)** 11-dehydrocorticosterone (11DHC) and **(D)** adrenocorticotropic hormone (ACTH). **(E–G)** The brain was also collected and the hippocampus was dissected for **(E)** corticosterone and **(F)** 11DHC quantification or **(G)** glucocorticoid-receptor (GR) expression. Data are presented as the means ± SEM of one experiment (*n* = 3–11 mice/genotype/group). Two-way analysis of variance (ANOVA) followed by Newman-Keuls Multiple Comparison Test, *p* < 0.05. *C57Bl/6 or 129*sv or Alox5*^−/−^ vs. CUS-C57Bl/6 or CUS-129*sv or* CUS-*Alox5*^−/−^, respectively; ^#^129*sv* vs. Alox5^−/−^; ^&^CUS-129*sv* vs. CUS-C57Bl/6 or CUS-*Alox5*^−/−^.

To determine the impact of 5-LO deficiency on stress-induced behavior and immune response, we began by comparing the amount of circulating corticosterone in two distinct mouse strains, C57Bl/6 and 129*sv*. The former strain has been widely characterized in studies addressing CUS-induced depression-like behavior (Pothion et al., 2004). Both mouse strains exhibited similar levels of serum corticosterone before CUS exposition (Figure 1A). Following 14 days of CUS exposure, serum levels of corticosterone increased significantly in both strains (*F*_(1,22)_ = 42.06, *p* < 0.001), but corticosterone levels remained comparable between them (Figure 1A). We observed similar blood neutrophil counts between both mouse strains at basal conditions, which were equally reduced after CUS exposition (*F*_(1,23)_ = 7.13, *p* < 0.02; Supplementary Figure S1B). Mononuclear cells were also equally represented in the blood of both strains at basal conditions, but these cells were significantly reduced in the blood of CUS-129*sv* compared to CUS-C57Bl/6 (interaction CUS vs. strain, *F*_(1,19)_ = 6.83, *p* = 0.017; Supplementary Figure S1A). Considering that 129*sv* mice submitted to CUS presented a similar profile in these parameters to that of C57Bl/6 mouse strain, we were confident to use the 129*sv* strain as controls of experiments performed with *Alox5*^−/−^ mice.

Deficiency of 5-LO abrogates the production of LTs and cysteinyl-LTs. Considering that HPA axis activation and corticosterone production are important components of the stress response, we sought to determine the impact of 5-LO deficiency on CUS-induced ACTH release in plasma samples, corticosterone production, and its metabolization into 11DHC in serum. At basal conditions, *Alox5*^−/−^ exhibited significant lower amounts of circulating corticosterone and 11DHC compared to 129sv mice (Corticosterone, *F*_(1,20)_ = 15.70, *p* < 0.001; 11DHC, *F*_(1,20)_ = 1239.0, *p* < 0.001; Figures 1B,C).We also found higher ACTH levels in *Alox5*^−/−^ mice (CUS vs. strain interaction; ACTH, *F*_(1,15)_ = 7.98, *p* = 0.013; Figure 1D). Compared to non-stressed 129*sv* controls, CUS-129*sv* mice produced 97% more circulating corticosterone and approximately 32% less circulating ACTH (Figures 1B,D). CUS-*Alox5*^−/−^ mice produced 87% more circulating corticosterone and had a reduction of approximately 64% in ACTH levels (Figures 1B,D). Together these results suggest that 5-LO deficiency results in lower corticosterone concentrations in the circulation, which triggers the activation of the HPA axis, reflected by high levels of plasma ACTH in *Alox5*^−/−^ mice at basal conditions. Furthermore, these data suggest similar hormonal profiles in the blood of *Alox5*^−/−^ mice at basal conditions and 129*sv* exposed to CUS.

Because depression affects primarily the brain, we investigated some aspects of stress response in the hippocampus of 129*sv* and *Alox5*^−/−^ mice. Both strains showed similar amounts of corticosterone (Figure 1E) and 11DHC (Figure 1F) at basal conditions. After CUS exposition, corticosterone levels equally increased in the hippocampus in both strains, but no changes were detected in 11DHC levels. Corticosterone levels are self-regulated by activation of GR. Thus, we hypothesized that the discrepancies between serum and hippocampus corticosterone levels in 129*sv* and *Alox5*^−/−^ mice could be related to lower GR expression, with consequent deregulation of the negative feedback loop in the HPA axis. We found that cells isolated from the hippocampus of non-stressed *Alox5*^−/−^ mice showed a trend to reduced GR expression (*p* = 0.08; Figure 1G). Importantly, hippocampal GR expression was significantly reduced in CUS-129*sv*, but not CUS-*Alox5*^−/−^ mice.

In addition, the number of circulating leukocyte was evaluated in the 129*sv* and *Alox5*^−/−^ mice exposed or unexposed to CUS. At a basal state, both strains presented similar numbers of circulating mononuclear cells, which decreased significantly after CUS (*F*_(1,20)_ = 196.1, *p* < 0.001; Supplementary Figure S1C). However, *Alox5*^−/−^ mice exhibited approximately 61% less neutrophils compared to 129*sv* at basal conditions (CUS vs. strain interaction, *F*_(1,20)_ = 20.16, *p* < 0.01; Supplementary Figure S1D). Interestingly, CUS exposure also reduced circulating neutrophil numbers by approximately 60% in 129*sv* mice, reaching similar levels to those of *Alox5*^−/−^ mice (Supplementary Figure S1D).

### *Alox5*^−/−^ Mice Have a Similar Profile of Stress-Induced Mediators to That of CUS-129*sv* Mice—Brain Structures Panorama

**Figure 2:**
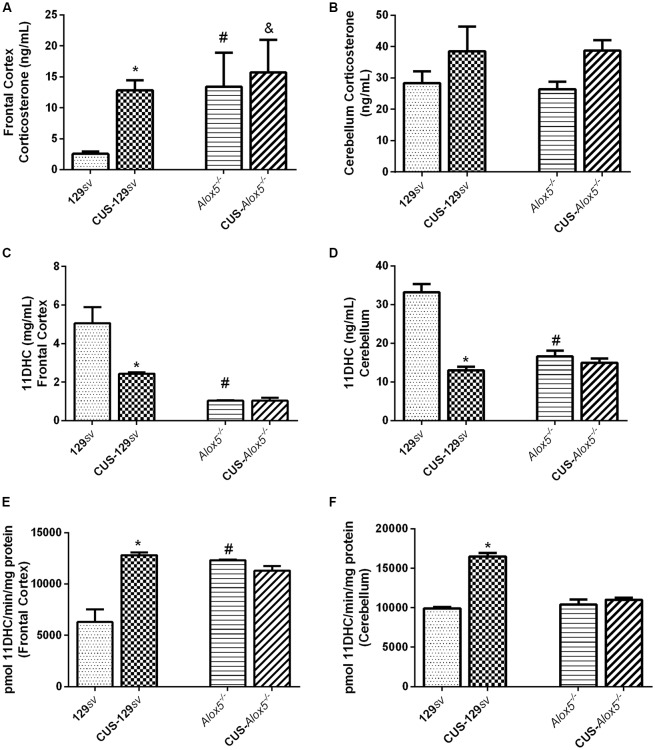
*Alox5*^−/−^ and CUS-129*sv* mice exhibit similar hormonal profile in different brain structures. 129*sv* and *Alox5*^−/−^ mice were subjected or not to CUS for 14 days. The animals were euthanized on the 15th day and the brains collected to dissect the frontal cortex and cerebellum. Quantification of **(A,B)** corticosterone **(C,D)** 11DHC and **(E,F)** 11β-hydroxysteroid dehydrogenase (11βHSD2) enzymatic activity in the frontal cortex and cerebellum, respectively. Data are presented as the means ± SEM of one experiment (*n* = 6–11 mice/genotype/group). Two-way ANOVA followed by Newman-Keuls Multiple Comparison Test, *p* < 0.05. *129*sv* vs. CUS-129*sv*; ^#^129*sv vs. Alox5*^−/−^; ^&^CUS-129*sv* vs. CUS-C57Bl/6 or CUS-*Alox5*^−/−^.

Contrasting data between blood and hippocampal corticosterone levels prompted us to perform a detailed investigation of different brain structures. Compared to 129*sv* mice, *Alox5*^−/−^ animals showed a significant increase in corticosterone production in the frontal cortex at basal conditions (Figure 2A). After CUS exposition, corticosterone levels increased by 420% in CUS-129*sv* mice compared to non-exposed 129*sv* animals and remained unaltered in *Alox5*^−/−^ mice (Figure 2A). As an additional negative control, we measured corticosterone concentrations in the cerebellum. As expected, there were no significant alterations on corticosterone levels in the cerebellum of 129*sv* or *Alox5*^−/−^ mice, neither before nor after CUS exposition (Figure 2B). However, *Alox5*^−/−^ mice presented lower 11DHC in the frontal cortex (*F*_(1,17)_ = 29.54, *p* < 0.0001; Figure 2C) and in the cerebellum (*F*_(1,17)_ = 20.41, *p* < 0.0003; Figure 2D) compared with 129*sv* mice. CUS exposure did not modify this profile. In contrast, CUS-129*sv* mice showed decreased 11DHC levels in both the frontal cortex (*F*_(1,17)_ = 6.873, *p* = 0.0179; Figure 2C) and cerebellum (*F*_(1,17)_ = 45.75, *p* < 0.0001; Figure 2D) when compared to 129*sv* animals.

The 11βHSD1 and 11βHSD2 enzymes are responsible for the metabolism of corticosterone, with 11βHSD1 promoting its production, whereas 11βHSD2 converts it into the inactive metabolite 11DHC. To investigate the potential molecular mechanism regulating the balance between corticosterone and 11DHC, we assessed the enzymatic activity of 11βHSD2 in the frontal cortex and cerebellum. Compared to 129*sv* mice, 11βHSD2 activity increased in the frontal cortex of *Alox5*^−/−^ and CUS-129*sv* mice (*F*_(1,17)_ = 8.237, *p* = 0.0106; *F*_(1,17)_ = 12.39, *p* = 0.0026; Figure 2E) and cerebellum of CUS-129*sv* (*F*_(1,17)_ = 85.16, *p* < 0.0001; Figure 2F). Despite the increased 11βHSD2 activity in the frontal cortex from CUS-129*sv* and in *Alox5*^−/−^ mice, the reduced 11DHC levels suggest that corticosterone is produced at a higher rate than consumed, as a result of a higher 11βHSD1 activity. It is also possible that corticosterone is catabolized by a different pathway that converts it into a metabolite other than 11DHC.

### *Alox5*^−/−^ Mice Exhibit a Natural Low Sucrose Preference at Basal Conditions—Behavior Panorama

**Figure 3:**
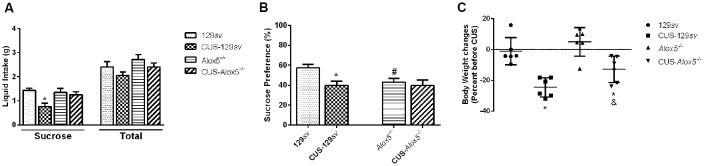
5-lipoxygenase (5-LO) deficiency results in decreased sucrose preference at basal conditions. 129*sv* and *Alox5*^−/−^ mice were exposed or not to CUS for a period of 14 days. Animals were deprived of water and food for 20 h on the 14th day. **(A,B)** On the 15th day, mice were evaluated for **(A)** liquid intake and **(B)** sucrose preference. **(C)** 129*sv* and *Alox5*^−/−^ mice were weighed on day 0, and exposed or not to CUS for a period of 14 days. Animals were weighed again on the 15th day, and the weight changes were expressed as a percentage before CUS. Data are presented as the means ± SEM and depict a representative experiment out of three independent experiments (*n* = 6–11 mice/genotype/group/experiment). Two-way ANOVA followed by Newman-Keuls Multiple Comparison Test, *p* < 0.05. *129*sv* vs. CUS-129*sv*; ^#^129*sv* vs. Alox5^−/−^.

We evaluated *Alox5*^−/−^ and 129*sv* strains, submitted or not to CUS, for sucrose preference, to determine the involvement of 5-LO deficiency on that behavior. The test assesses the reduction in sugar solution intake and sucrose preference in relation to a control group (Guo et al., 2009). Exposure to CUS decreased sucrose solution, but not total liquid intake by CUS-129*sv* mice compared to 129*sv* animals in basal conditions (CUS, *F*_(1,36)_ = 8.10, *p* = 0.007, CUS vs. strain, *F*_(1,36)_ = 4.68, *p* = 0.03; Figure 3A). This was reflected by lower sucrose preference (*F*_(3,36)_ = 5.11, *p* = 0.005; Figure 3B), indicating that 129*sv* mice, which express 5-LO, develop decreased sucrose preference when exposed to CUS. However, compared to non-stressed 129*sv* mice, *Alox5*^−/−^ animals at basal conditions already exhibited lower sucrose preference, which remained unchanged after 14 days of CUS exposure (Figure 3B). These results demonstrate that the *Alox5*^−/−^ mice present a natural lower sucrose preference, which is comparable to 129*sv* mice exposed to CUS. Furthermore, that behavior was not altered in *Alox5*^−/−^ mice. Therefore, impaired LTB_4_ production could predispose these animals to reach a maximal decreased sucrose preference response that remains unchanged even under conditions of chronic stress. We also weighed the whole body of 129*sv* and *Alox5*^−/−^ before and after 14 days of CUS (Figure 3C).CUS exposure significantly reduced the body weight in both mouse strains (*F*_(1,20)_ = 8.41, *p* < 0.01). Supporting our previous findings, these results confirm a significant impact of 5-LO deficiency in a natural depressive-like phenotype of *Alox5*^−/−^ mice that remains mostly unaltered after stressful stimuli.

### 5-LO Deficiency Impacts the Production of Inflammatory Mediators Before and After CUS—Spleen Panorama

**Figure 4:**
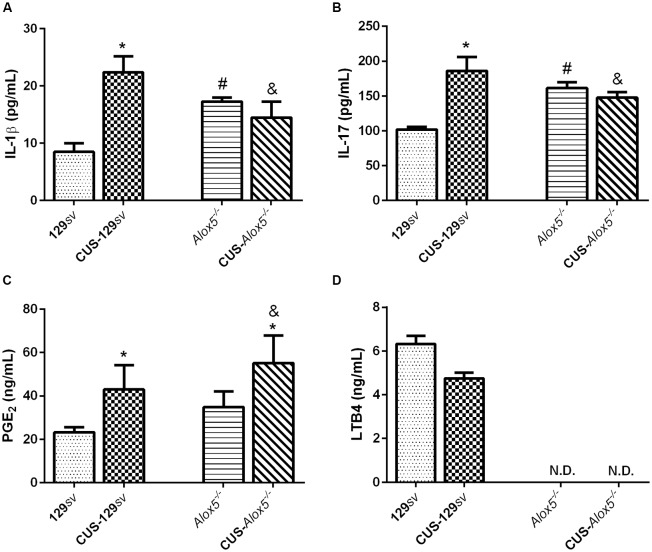
5-LO regulates the production of inflammatory mediators before and after CUS exposition. 129*sv* and *Alox5*^−/−^ mice were exposed or not to CUS for 14 days. Animals were euthanized on the 15th days and spleens were collected for quantification of **(A)** IL-1β, **(B)** IL-17, **(C)** PGE_2_ and **(D)** leukotrienes B_4_ (LTB_4_). Data are presented as means ± SEM of one representative out of two independent experiments (*n* = 6 mice/genotype/group/experiment). Two-way ANOVA followed by Newman-Keuls Multiple Comparison Test, *p* < 0.05. *129*sv* or *Alox5*^−/−^ vs. CUS-129*sv* or CUS-*Alox5*^−/−^; ^#^129*sv* vs. Alox5^−/−^; ^&^CUS-129*sv* vs. CUS-*Alox5*^−/−^. ND, not determined.

Inflammatory mediators such as IL-1β, IL-17 and PGE_2_ mediate stress responses (Müller et al., 2006; Goshen and Yirmiya, 2009; Guo et al., 2009; Beurel et al., 2013), while 5-LO and LTs play significant roles during inflammatory processes (Filgueiras et al., 2015; Zoccal et al., 2018). Using a model of sterile inflammation, we have shown previously that *Alox5*^−/−^ mice produce higher amounts of IL-1β and PGE_2_ compared to 129*sv* mice (Zoccal et al., 2016). In view of the natural depressive-like behavior of *Alox5*^−/−^ mice, we thus investigated whether this phenotype correlates with alterations on the splenic production of inflammatory mediators by both mice strains exposed or not to CUS. Compared 129*sv* mice at basal conditions, levels of IL-1β, IL-17 and PGE_2_ increased by 164% (*F*_(1,17)_ = 6.06, *p* = 0.025; Figure 4A), 83% (*F*_(1,25)_ = 11.61, *p* = 0.002; Figure 4B), and 85% (*F*_(1,25)_ = 5.46, *p* = 0.028; Figure 4C) in the spleen of CUS-129*sv* animals, respectively. In addition, LTB_4_ concentration decreased by almost 25% (*t-test*, *p* = 0.054; Figure 4D). IL-1β, IL-17, and PGE_2_ levels were also elevated in the spleen of *Alox5*^−/−^ mice by 104%, 59%, and 50% compared to 129*sv* animals at basal conditions, respectively (Figures 4A–C). However, IL-1β and IL-17 levels were significantly reduced in the spleen of *Alox5*^−/−^ mice both after CUS exposition, compared to CUS-129*sv* animals (Figures 4A,B). In contrast to all phenomena described so far, PGE_2_ increased significantly in the spleen of *Alox5*^−/−^ mice exposed to CUS when compared to non-stressed *Alox5*^−/−^ or even CUS-129*sv* (Figures 4A–C).

### *Alox5*^−/−^ Mice at Basal Conditions Exhibit Stress-Like Profiles of Caspase-1 Expression and 11βHSD2 Activity—Spleen Panorama

**Figure 5:**
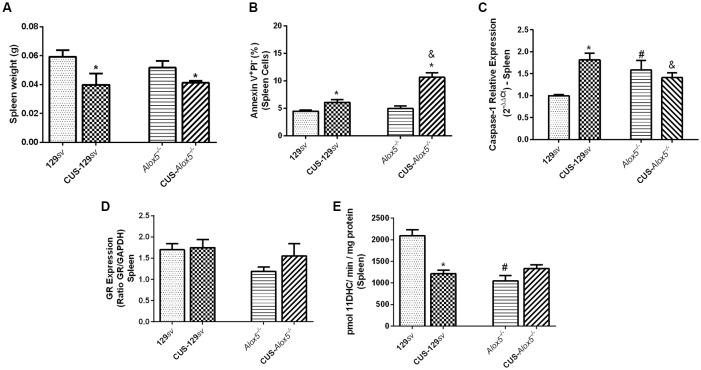
Spleens from *Alox5*^−/−^ mice have increased caspase-1 expression, but lower 11βHSD2 activity compared to 129*sv* at basal conditions. Both 129*sv* and *Alox5*^−/−^ mouse strains were subjected or not to CUS for 14 days. Animals were euthanized on the 15th day and the spleen was collected. **(A)** Spleen weighs before and after CUS exposition. **(B)** Apoptosis assay measured by flow cytometry. **(C)** Transcriptional caspase-1 expression. **(D)** Protein GR expression. **(E)** 11βHSD2 activity. Data are presented as means ± SEM and represent one out of two independent experiments (*n* = 6 mice/genotype/group/experiment). Two-way ANOVA followed by Newman-Keuls Multiple Comparison Test, *p* < 0.05. *129*sv* or *Alox5*^−/−^ vs. CUS-129*sv* or CUS-*Alox5*^−/−^; ^#^129*sv* vs. *Alox5*^−/−^; ^&^CUS-129*sv* vs. CUS-*Alox5*^−/−^.

Both 129*sv* and *Alox5*^−/−^ mice showed reduced spleen weights after CUS exposition (Figure 5A), explained by increased apoptosis of their splenic cells (*F*_(1,11)_ = 27.7, *p* < 0.001; Figure 5B), which was more pronounced in the spleens of CUS-*Alox5*^−/−^ mice (CUS vs. strain, *F*_(1,11)_ = 18.6, *p* < 0.001). Compared to 129*sv* mice, *Alox5*^−/−^ animals at basal conditions exhibited higher amounts of splenic IL-1β (Figure 4A). Caspase-1 activation largely drives IL-1β maturation and release (Friedlander et al., 1996; Rano et al., 1997). We thus predicted that caspase-1 expression would be increased in spleens of *Alox5*^−/−^ mice at basal conditions. As expected, the deficiency in 5-LO related metabolites results in higher caspase-1 expression compared to non-stressed 129*sv* mice (CUS vs. strain, *F*_(1,8)_ = 35.94; Figure 5C). Curiously, caspase-1 expression also increased in CUS-129*sv* mice compared to non-stressed 129*sv* animals but remained equally expressed in *Alox5*^−/−^ mice even after CUS exposition (Figure 5C).

Paugh et al. (2015) demonstrated that GR can be cleaved by caspase-1, resulting in glucocorticoid resistance. Systemic corticosterone concentration is self-regulated by GR activation, whereas 5-LO deficiency induces lower levels of circulating corticosterone. We thus hypothesized that lower GR expression would explain this reduction. However, there was no significant change in GR expression between both mouse strains before or after CUS exposition (Figure 5D). Due to its role in corticosterone degradation, we also assessed 11βHSD2 activity, which was significantly reduced in the spleens of *Alox5*^−/−^ mice (*F*_(1,17)_ = 17.09, *p* = 0.0007) and in CUS-129*sv* mice (*F*_(1,17)_ = 7.143, *p* = 0.0161) compared with 129*sv* (Figure 5E). Together, our results indicate that 5-LO derived metabolites regulate the apoptotic cellular death in the spleen, which associates with increased caspase-1 expression and decreased 11βHSD2 activity at basal conditions, that remains at the same levels after chronic stress.

### *Alox5*^−/−^ Mice at Basal Conditions Exhibit Stress-Like Profiles of Caspase-1 Expression and 11βHSD2 Activity—Adrenal Panorama

**Figure 6:**
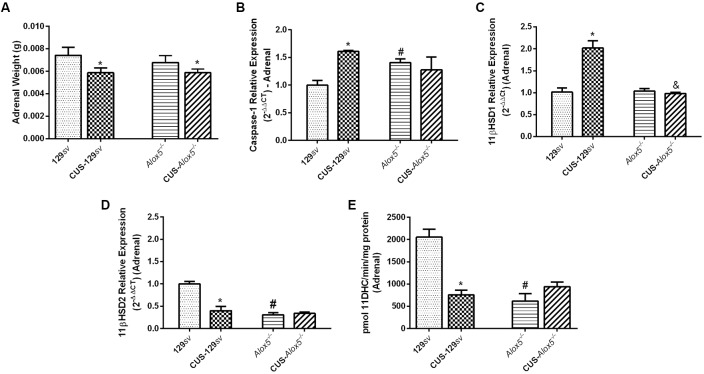
Adrenal glands from *Alox5*^−/−^ mice have increased caspase-1 expression, but lower 11βHSD2 activity compared to 129*sv* at basal conditions. 129*sv* and *Alox5*^−/−^ mice were subjected or not to CUS for 14 days. Animals were euthanized on the 15th days and the adrenal glands were collected. **(A)** Adrenal gland weight before and after CUS exposition. **(B)** Transcriptional expression of caspase-1, **(C)** 11βHSD1 and **(D)** 11βHSD2 in adrenal glands. **(E)** Enzymatic 11βHSD2 activity in adrenal glands. Data are presented as means ± SEM and represent one of two independent experiments (*n* = 6 mice/genotype/group/experiment). Two-way ANOVA followed by Newman-Keuls Multiple Comparison Test, *p* < 0.05. *129*sv* or *Alox5*^−/−^ vs. CUS-129*sv* or CUS-*Alox5*^−/−^; ^#^129*sv* vs. *Alox5*^−/−^; ^&^CUS-129*sv* vs. CUS-*Alox5*^−/−^.

The HPA axis showed increased activation in *Alox5*^−/−^ mice at basal conditions. We, therefore, investigated the presence of possible molecular changes in the adrenal glands in both mouse strains exposed or not to CUS. As observed for the spleens, CUS exposure reduced adrenal gland weight in both strains (Figure 6A). Caspase-1 expression also increased in adrenal glands of CUS-129*sv* and *Alox5*^−/−^ mice at basal conditions (*F*_(1,6)_ = 15.63 *p* < 0.01; Figure 6B). In this situation, the adrenal glands of both 129*sv* and *Alox5*^−/−^ strains expressed similar levels of 11βHSD1, which remained equally expressed in CUS- *Alox5*^−/−^, but increased significantly in CUS-129sv mice (CUS vs. strain, *F*_(1,16)_ = 27.32, *p* < 0.001; Figure 6C). Compared to 129*sv* mice at basal conditions, CUS-129*sv* and *Alox5*^−/−^ animals expressed significantly less 11βHSD2 (CUS vs. strain, *F*_(1,7)_ = 22.26, *p* < 0.001; Figure 6D). Of note, CUS exposure of *Alox5*^−/−^ does not seem to cause a significant impact on the expression of any of these enzymes in the adrenal gland (Figures 6C,D). Importantly, at basal conditions *Alox5*^−/−^ and CUS-129*sv* mice exhibited lower 11βHSD2 enzymatic activity compared to 129*sv* mice (*F*_(1,17)_ = 17.81, *p* = 0.0006; *F*_(1,17)_ = 10.85, *p* = 0.0043 respectively; Figure 6E).

### LTB_4_ Regulates Levels of Systemic Corticosterone and Caspase-1 Expression

**Figure 7:**
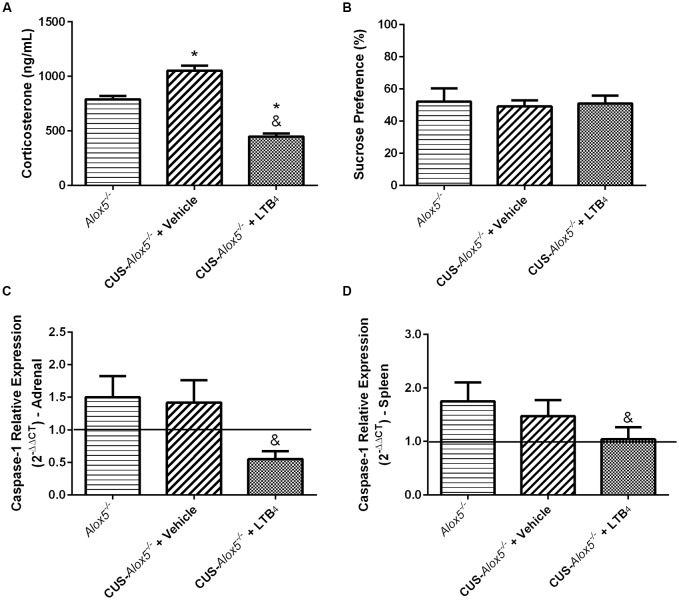
Exogenous LTB_4_ decreases corticosterone production and caspase-1 expression, but not decreased sucrose preference in *Alox5*^−/−^ mice. Animals were subjected or not to CUS over a period of 14 days. CUS-*Alox5*^−/−^ mice were treated twice a day (12 h/12 h) with LTB_4_ (50 ng/animal/100 μL i.p.) or vehicle (100 μL i.p.). **(A)** Blood was collected on the 15th day of quantification of circulating corticosterone. **(B)** Before euthanasia on the 15th day, mice were evaluated for sucrose preference. **(C)** Adrenal glands and **(D)** spleens were removed for evaluation of the transcriptional caspase-1 expression. In **(C,D)**, 129*sv* mice at basal conditions were used as a reference (continuous line), and *Alox5*^−/−^ mice at basal conditions were used as controls. Data are presented as means ± SEM of one experiment with *n* = 6–8 mice/genotype/group. Two-way ANOVA followed by Newman-Keuls Multiple Comparison Test, *p* < 0.05. *Alox5^−/−^ vs. CUS-*Alox5*^−/−^ + Vehicle or CUS-*Alox5*^−/−^ + LTB_4_; ^&^CUS-*Alox5*^−/−^ + Vehicle vs. CUS-*Alox5*^−/−^ + LTB_4_.

At this point of our study, we have demonstrated that *Alox5*^−/−^ mice exhibit depressive-like phenotype at basal conditions. Excluding PGE_2_ production, CUS exposure does not seem to cause a significant impact on this profile, which is very similar to that of CUS-129*sv* mice. Considering that *Alox5*^−/−^ animals lack all 5-LO-derived metabolites and LTB_4_ is the major product of this pathway (Samuelsson, 2000), we performed a pharmacological treatment with this lipid mediator to evaluate its specific involvement on the altered biological and behavioral responses due to 5-LO deficiency (Supplementary Figure S2). Interestingly, LTB_4_ reduced serum corticosterone levels of CUS-*Alox5*^−/−^ mice compared to both *Alox5*^−/−^ and CUS-*Alox5*^−/−^ mice at basal conditions treated with vehicle (Figure 7A). In contrast, all groups exhibited similar sucrose preference (Figure 7B). Together, these results suggest that, at least in this model, corticosterone does not mediate the decreased sucrose preference. We have shown previously that LTB_4_ inhibits IL-1β release during sterile inflammation (Zoccal et al., 2016, 2018). Therefore, we evaluated whether this change was associated with the levels of caspase-1 expression. Compared to vehicle-injected CUS-*Alox5*^−/−^ mice, LTB_4_ treatment significantly decreased caspase-1 expression in the adrenal glands (*F*_(2,8)_ = 10.52, *p* < 0.06; Figure 7C) and in the spleen (*F*_(2,12)_ = 5.91, *p* = 0.016; Figure 7D). These results could help to explain the high levels of IL-1β production of *Alox5*^−/−^ mice (Figure 4A). Taken together, our results indicate that, besides inhibiting intracellular cAMP production (Zoccal et al., 2016, 2018), LTB_4_ also inhibits caspase-1 expression to suppress IL-1β release. Moreover, these data demonstrate that LTB_4_ also regulates the levels of systemic corticosterone production after CUS exposure.

## Discussion

5-lipooxygenase (5-LO), encoded by the *Alox5* gene, catalyzes the formation of (LTB_4_) which is important for the regulation of the HPA-axis and consequently corticosterone levels. This study showed that conditional *Alox5*^−/−^ mice present reduced sucrose preference, suggesting reduced reward sensitivity. Furthermore, caspase-1, IL-1β, IL-17 and PGE_2_ increased in these animals. In frontal cortex corticosterone also increased, although its systemic levels decreased. The observed alterations were in general similar to those observed in the CUS-induced changes in control animals. In this case, however, systemic corticosterone levels increased and, notably, LTB_4_ levels decreased. LTB_4_ administration restored caspase-1 levels and systemic corticosterone levels, but not reduced sucrose preference.

In the past few years, several reports have provided evidence for the direct relationship between depression and inflammation. However, the role of 5-LO-derived metabolites in depression onset and progression is unclear (Uz et al., 2008; Athayde et al., 2009; Chu and Praticò, 2009; Joshi and Praticò, 2013; Joshi et al., 2015). LTB_4_ is the main product of 5-LO activation. CUS exposure of 129*sv* mice, however, had a modest impact over LTB_4_ production in the spleen (*p* = 0.054). In line with these findings, reduced (CUS-129*sv*) or absence (*Alox5*^−/−^) LTB_4_ production influenced several parameters associated with stress-induced behavior, although we cannot exclude the influence of other 5-LO related metabolites. *Alox5*^−/−^ animals at basal conditions already exhibited lower interest for sucrose. We also detected, using the tail suspension test (TST), that *Alox5*^−/−^ mice exhibited a natural depressive-like behavior, assessed through the immobility induced by their inability or reluctance to maintain effort rather than a generalized hypoactivity. CUS exposition, however, did not change that behavior. Besides that, we observed that CUS exposure did not cause a significant impact on the immobility time of 129*sv*, indicating that 14 days of CUS exposure were not enough to change this behavior, contrasting with sucrose preference (data not shown). Furthermore, exposure to CUS did not impact any of the evaluated behavioral parameters in *Alox5*^−/−^ mice, which were, however, similar to those observed in CUS-129*sv* animals. This finding shows that 5-LO-deficiency results in decreased sucrose preference that is not modified by chronic stress, possibly indicating a ceiling effect. One limitation of our study is that 129*sv* and *Alox5*^−/−^ mice were not littermates, not sharing, therefore, the same intra-uterine and preweaning environment. This limitation is relevant because the maternal behavior could have been affected by 5-LO deletion and, consequently, affected the phenotype of the adult offspring (Gingrich and Hen, 2000). Although the pharmacological results, discussed above, suggest that even this possibility exists, the involvement of LTB_4_ in the observed behavioral changes is likely. Future studies are necessary to confirm whether the phenotypes observed here were replicated when 129*sv* and *Alox5*^−/−^ were born and weaned by the same mother.

Of importance, *Alox5*^−/−^ mice at basal conditions displayed increased levels of circulating ACTH, indicating that 5-LO deficiency triggers the HPA axis activation even in the absence of stressful stimuli. However, systemic levels of corticosterone were reduced in *Alox5*^−/−^ mice at basal conditions. They increased after exposure to CUS but did not reach the same levels of CUS-129*sv* mice. Therefore, our data corroborate the proposal that 5-LO and related metabolites regulate the activity of HPA axis (Joshi and Praticò, 2013). We hypothesized that 11βHSD activity would be associated with the reduced levels of circulating corticosterone. To test this, we investigated the expression and/or activity of 11βHSD isoforms. The regulation of 11βHSD1 and 11βHSD2 expression is a complex process that directly controls corticosterone levels. Interestingly, IL-1β increases the expression of 11βHSD1 (Tetsuka et al., 1999), while reducing the expression and activity of 11βHSD2 (Chisaka et al., 2005). In line with these facts, our results show that CUS-129*sv* mice presented increased IL-1β levels and 11βHSD1 expression in the spleen, concomitantly with reduced 11βHSD2 expression. The reduced activity of 11βHSD2 in their spleens or adrenal glands, however, suggest that 5-LO and related metabolites engage distinct molecular mechanisms to regulate levels of systemic corticosterone.

Contrasting with the spleen and adrenal glands, the frontal cortex of *Alox5*^−/−^ and CUS-129*sv* mice exhibited increased 11βHSD2 activity. These discrepancies suggest that 5-LO deficiency or CUS exposition results in organ-dependent changes. Future studies are needed to elucidate the mechanisms underlying such differences.

The basal inflammatory state of an individual is highly relevant in the context depression (Maes et al., 1997). We demonstrated that *Alox5*^−/−^ mice at basal conditions already exhibit increased levels of splenic IL-1β and IL-17, which are associated with behavioral changes. They also develop exacerbated inflammatory responses and become highly susceptible to a model of sterile inflammation (Zoccal et al., 2016), suggesting that the molecular mechanisms underlying the behavioral modifications of *Alox5*^−/−^ mice at basal conditions are directly involved with a deregulated basal inflammatory profile. Thus, non-stressed *Alox5*^−/−^ animals could already release cytokines at high levels (i.e., the *ceiling effect*), justifying why stress did not cause a further increase of these mediators.

Strikingly, we found that CUS exposition results in increased PGE_2_ production, whereby CUS-*Alox5*^−/−^ mice exhibited even higher levels compared to CUS-129*sv*. We have demonstrated that PGE_2_ boosts IL-1β production by innate immune cells (Zoccal et al., 2016, 2018). The release of this cytokine is conditioned to the activation of inflammatory caspases (Friedlander et al., 1996; Rano et al., 1997). Higher levels of PGE_2_ were associated with increased caspase-1 expression in basal conditions of *Alox5*^−/−^ and CUS-129*sv* mice, suggesting that, beyond PGE_2_ signaling, LTB_4_ also represses caspase-1 expression to limit IL-1β release. Paugh et al. (2015) demonstrated that higher caspase-1 expression augments GR cleavage, resulting in elevated glucocorticoid resistance. GR regulation is an important checkpoint during corticosterone production. Of note, GR expression was significantly reduced in the hippocampus of CUS-129*sv*, suggesting that levels of systemic corticosterone increase due a disruption on negative feedback, which was not observed in *Alox5*^−/−^. Despite the increased circulating levels of ACTH in *Alox5*^−/−^ mice, they exhibited reduced corticosterone levels. These levels increased after CUS but remained significantly lower compared to CUS-129*sv* mice. Thus, 5-LO and related metabolites might regulate the systemic levels of corticosterone by promoting the expression and signaling of the ACTH receptor (MC2R) in the adrenal glands. However, future studies are needed to test this hypothesis.

Compared to *Alox5*^−/−^ mice at basal conditions, CUS-*Alox5*^−/−^ show increased levels of systemic corticosterone. Treatment with LTB_4_ reduced circulating corticosterone but did not cause any alteration on sucrose preference. Considering that *Alox5*^−/−^ mice are unable to produce other molecules beyond LTB_4_, we cannot discard the involvement and synergy between LTC_4_, D_4_ or E_4_ in sucrose preference behavior. A recent study demonstrated that the knockdown if the cysteinyl-LT receptor 1 in the hippocampus reverses the depression-like behavior of mice submitted to CMS (Yu et al., 2016). LTB_4_ treatment was unable to reduce the decreased sucrose preference, but regulated caspase-1 expression, IL-1β release, and corticosterone production. These findings suggest that, while LTB_4_ controls the production of mediators involved in the inflammatory response, decreased sucrose preference could be directly regulated by cysteinyl-LTs. Further investigation will reveal the real contribution of these cysteinyl-LTs and LTB_4_ to the onset and progression of depression. Patients with major depressive disorders have lower levels of eicosanoid precursors, the polyunsaturated fatty acids (PUFA), which is associated with the severity of depressive symptoms (Adams et al., 1996). Antidepressant effects in these patients might involve eicosanoid cascades such as PGE_2_ formation (Su, 2009). Drugs such as imipramine, desipramine, and clomipramine reduce systemic levels of corticosterone in rats submitted to force swimming stress test, which prolonged swimming time (Yamada et al., 1987). Similar to LTB_4_ treatment, several antidepressants reduced caspase-1 expression and IL-1β production (Alcocer-Gómez et al., 2017). While LTB_4_ treatment did not affect sucrose preference, decreased inflammation induced by the treatment with LTB_4_ or antidepressants might account for changes in other behavioral parameters. A limitation of this study is that there was no group treated with antidepressant. Therefore, future studies comparing the effects of LTB_4_ and antidepressants in 129*sv* and *Alox5*^−/−^ mice are needed to address this problem.

A previous work demonstrated that exogenous administration of LTB_4_ activates the HPA axis *via* BLT1 receptor, increasing plasma levels of ACTH and corticosterone while downregulating the inflammatory response during asthma (Zhang et al., 2010). However, our study is the first to demonstrate that 5-LO and related metabolites promote differential regulation of the systemic levels of ACTH and corticosterone, impacting stress and inflammation. This is highly relevant to explain the controversial findings of the role of LTB_4_ in infectious diseases. It is well recognized that LTB_4_ increases phagocytosis of microorganisms *in vitro* (Serezani et al., 2005; Secatto et al., 2014; Prado et al., 2017), as does dexamethasone (van der Goes et al., 2000). However, the results regarding LTB_4_ function *in vivo* are conflicting. For example, different outcomes have been described for experimental tuberculosis using LTB_4_ pharmacological inhibition or gene deletion. Indeed, treatment with MK886 inhibits LTB_4_ production and increases bacterial burden and mortality of mice infected with *Mycobacterium tuberculosis* (Peres et al., 2007). In contrast, *Alox5*^−/−^ mice are more resistant to *M. tuberculosis* infection, have lower bacterial burden, and survive longer than 129*sv* animals (Bafica et al., 2005). These opposing results generate a paradox regarding the importance of LTB_4_ in the control of long-lived bacterial infections such as *M. tuberculosis*. Based on our present data, we suggest that the low levels of corticosterone observed in *Alox5*^−/−^ mice favors the host immune response by promoting a quick pathogen elimination. Although major depression is a polygenic condition and depends on the interaction with environmental factors (Kendler et al., 1995), the results indicate that a decrease in 5-LO expression could be associated with this disorder.

**Figure 8:**
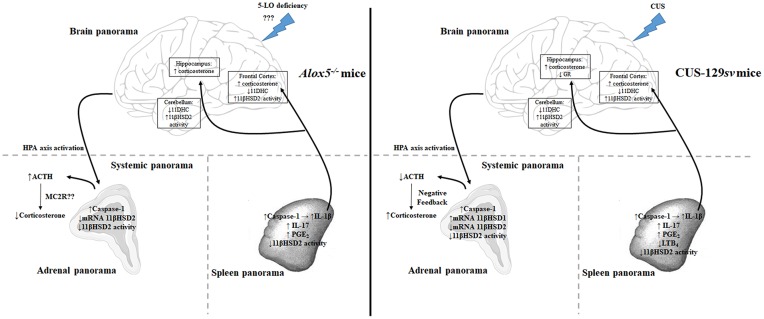
*Alox5*^−/−^ mice display a depressed-like behavior at basal conditions comparable to that of 129*sv* animals exposed to CUS. In general, corticosterone levels and 11βHSD2 activity increased and 11DHC levels decreased in the brains of *Alox5*^−/−^ and CUS-129*sv* mice. These animals also exhibited increased caspase-1 expression in the adrenal gland and spleen associated with increased levels of splenic IL-1β. In addition, 5-LO deficiency at basal conditions or CUS exposure of 129*sv* mice decreased 11βHSD2 mRNA expression and/or activity in the adrenal gland and spleen, while increasing IL-17 and PGE_2_ or decreasing LTB_4_ production in the spleen. However, 5-LO deficiency culminates in elevated basal activation of the HPA axis, reflected by high levels of circulating ACTH but reduced levels of systemic corticosterone, possibly due to the impaired expression of the ACTH receptor (MC2R) in the adrenal glands (systemic panorama—left panel). In contrast, CUS-129*sv* mice exhibited reduced circulating levels of ACTH and increased corticosterone (systemic panorama—right panel) and decreased GR expression in the hippocampus (brain panorama—right panel).

In summary (Figure 8; Supplementary Table S1), we demonstrated that CUS results in significant modifications in 129*sv* mice, increasing corticosterone levels and reducing GR expression in hippocampus. In addition, the HPA axis is activated, resulting in increased circulating corticosterone and a series of other organ responses, such as increased caspase-1 expression in the spleen and adrenal glands; increased levels of splenic 11βHSD1 expression and IL-1β and PGE_2_ production; decreased levels of splenic LTB_4_ and 11βHSD2 expression. Basically, the same phenomena occur in *Alox5*^−/−^ mice at basal conditions. 5-LO and related metabolites control caspase-1 expression, IL-1β, and PGE_2_ release. This inflammatory environment possibly increases HPA activation. The adrenal glands, however, seem to be unable to produce enough corticosterone, which results in lower circulating amounts of this hormone, even after CUS exposition.

In conclusion, this study provides important insights into the role of 5-LO and related metabolites in the control of stress-induced mediators and inflammation. suggesting that *Alox5*^−/−^ mice could be a novel model for the understanding of the molecular mechanisms responsible for decreased sensitivity to rewards.

## Ethics Statement

This study was carried out in accordance with the recommendations of National Council for Control of Animal Experimentation (CONCEA). The protocol was approved by the Ethics Committee on Animal Use (CEUA) of the Campus of Ribeirão Preto (Protocol number: 14.1.290.53.5).

## Author Contributions

GL designed the research, analyzed the data and prepared the figures. GL and MP executed the main experimental procedures and wrote the article. KZ, PP, MB, and AP assisted with laboratory procedures. MF assisted with initial CUS protocol. LF, CS, EDB and FG analyzed the data and discussed the hypothesis. FG performed statistical analysis. LF conceived and supervised the project, designed the experiments, obtained the funding grants, helped with the data interpretation, wrote and discussed the manuscript and acted as scientific advisor.

## Conflict of Interest Statement

The authors declare that the research was conducted in the absence of any commercial or financial relationships that could be construed as a potential conflict of interest.

## Footnotes

^1^http://rsb.info.nih.gov
