# Supplementary material for: Paradoxical Effect of LTB4 on the Regulation of Stress-Induced Corticosterone Production
Source: Front Behav Neurosci. 2019 Apr 16;13:73. doi: 10.3389/fnbeh.2019.00073 (PMC6477085; doi:10.3389/fnbeh.2019.00073)
Supplement: Supplementary file 1 [file Data_Sheet_1.PDF]

**SUPPLEMENTARY DATA**

# Paradoxical effect of LTB4 on the regulation of stress-induced corticosterone production

Gisele A. Locachevic[1†], Morgana K. B. Prado[1†], Karina F. Zoccal[1], Priscilla A. T. Pereira[1], Carlos A. Sorgi[1], Mariza Bortolanza[2], Ana Paula F. Peti[1], Manoela V. Fogaça[3], Francisco S. Guimarães[3], Elaine Del Bel[2], Lúcia H. Faccioli[1*].

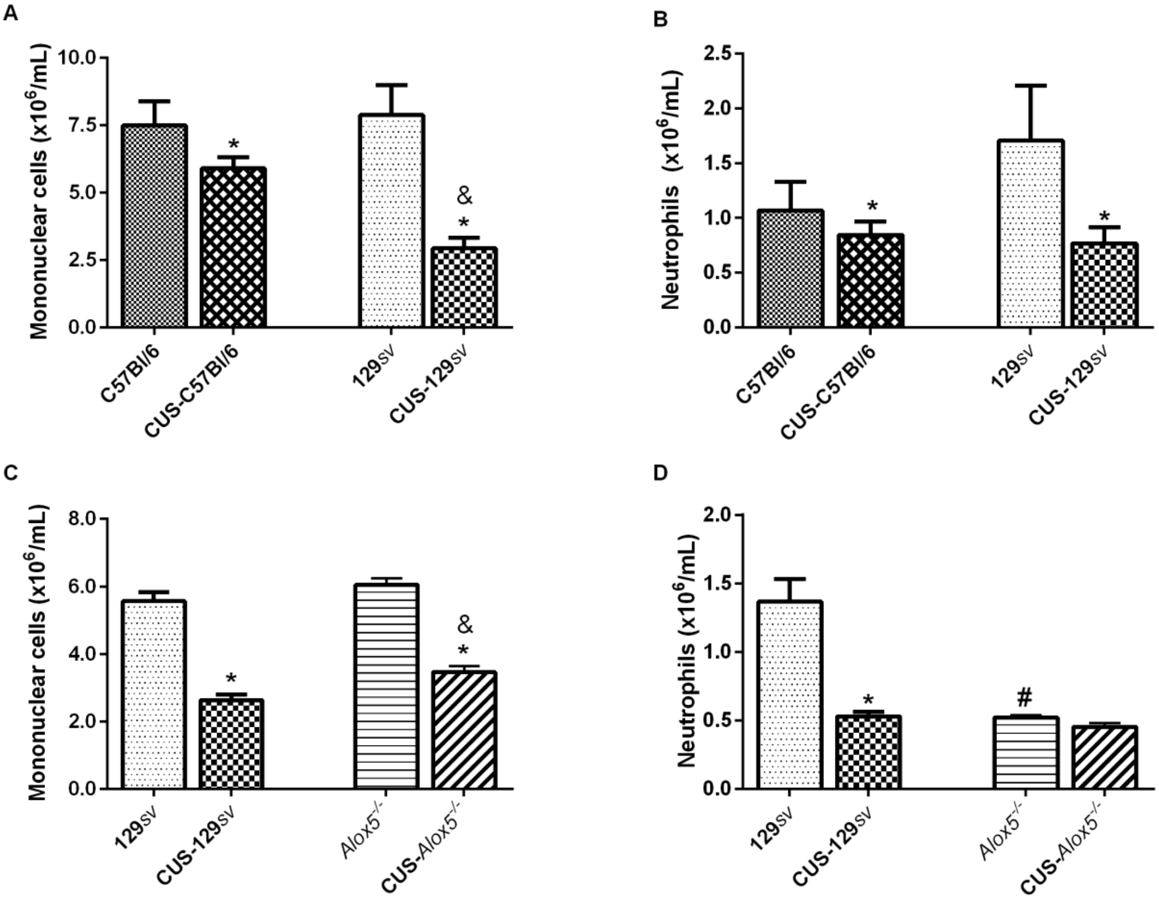


**Figure S1.** 129*sv* and C57Bl/6 mice exhibit similar cellular profiles after CUS exposure, whereas 5-LO deficiency impacts neutrophil counts**.** The C57Bl/6, 129*sv* and *Alox5*$^{-/-}$ mice were subjected or not to CUS for fourteen days. Animals were euthanized on the 15$^{th}$ day and the blood was collected for (**A, C**) mononuclear cell and (**B, D**) neutrophil counting. Data are presented as the means ± SEM of one experiment (n = 3-10 mice/genotype/group). Two-way ANOVA followed by Newman-Keuls Multiple Comparison Test, $p < 0.05$. *C57Bl/6 or 129*sv* or *Alox5*$^{-/-}$ *vs* CUS-C57Bl/6 or CUS-129*sv* or CUS-*Alox5*$^{-/-}$, respectively; $^{\#}$129*sv vs Alox5*$^{-/-}$; $^{\&}$CUS-129*sv vs* CUS-C57Bl/6 or CUS-*Alox5*$^{-/-}$.

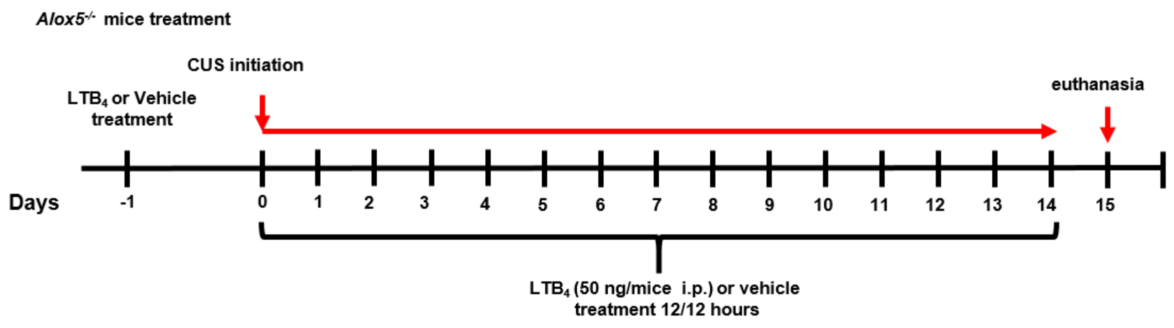


**Figure S2**. Scheme for $LTB_4$ treatment. *Alox5*$^{-/-}$ mice were left untreated or treated with vehicle or $LTB_4$ (50 ng/mice, i.p.) 24 h before being subjected to stress, and again every 12 h over a period of fourteen days, which represents the CUS exposure period. At the same time points, control mice received the vehicle used to dilute the $LTB_4$.
