# Supplementary material for: Paradoxical Effect of LTB4 on the Regulation of Stress-Induced Corticosterone Production
Source: Front Behav Neurosci. 2019 Apr 16;13:73. doi: 10.3389/fnbeh.2019.00073 (PMC6477085; doi:10.3389/fnbeh.2019.00073)
Supplement: Supplementary file 2 [file Table_1.DOC]

**Table S1.** Summary of the main differences between 129*sv* and *Alox5^-/-^* mice and responses observed after CUS exposure.

|  | | | | |
| --- | --- | --- | --- | --- |
| **Parameters** | **129*sv*** | **CUS-129*sv*** | ***Alo5x^-/-^*** | **CUS-*Alox5^-/-^*** |
| Sucrose Preference | S | ↓ ^(129^*^sv^*) | ↓ ^(129^*^sv^*) | = (*^Alox5-/-^*^; CUS-129^*^sv^*) |
| Circulating Corticosterone | S | ↑ ^(129^*^sv^*) | ↓ ^(129^*^sv^*) | ↑ (*^Alox5-/-^*) ; ↓ ^(CUS-129^*^sv^*) |
| Frontal Cortex Corticosterone | S | ↑ ^(129^*^sv^*) | ↑ ^(129^*^sv^*) | = (*^Alox5-/-^*) |
| ACTH | S | ↓ ^(129^*^sv^*) | ↑ ^(129^*^sv^*) | ↑ (*^Alox5-/-^*) ; ↓ ^(CUS-129^*^sv^*) |
| IL-1β | S | ↑ ^(129^*^sv^*) | ↑ ^(129^*^sv^*) | = (*^Alox5-/-^*) ; ↓ ^(CUS-129^*^sv^*) |
| IL-17 | S | ↑ ^(129^*^sv^*) | ↑ ^(129^*^sv^*) | = (*^Alox5-/-^*) ; ↓ ^(CUS-129^*^sv^*) |
| PGE_2_ | S | ↑ ^(129^*^sv^*) | = ^(129^*^sv^*) | ↑ (*^Alox5-/-^*^; CUS-129^*^sv^*) |
| LTB_4_ | S | Trend to ↓ ^(129^*^sv^*) (p=0.054) | N.D. | N.D. |
| mRNA 11βHSD1 | S | ↑ ^(129^*^sv^*) | = ^(129^*^sv^*) | = (*^Alox5-/-^*) ; ↓ ^(CUS-129^*^sv^*) |
| mRNA 11βHSD2 | S | ↓ ^(129^*^sv^*) | ↓ ^(129^*^sv^*) | = (*^Alox5-/-^*^; CUS-129^*^sv^*) |
| 11βHSD2 activity (spleen and adrenal) | S | ↓ ^(129^*^sv^*) | ↓ ^(129^*^sv^*) | = (*^Alox5-/-^*^; CUS-129^*^sv^*) |
| 11βHSD2 activity (F.C. and cerebellum) | S | ↑ ^(129^*^sv^*) | ↑F.C. ^(129^*^sv^*) | = (*^Alox5-/-^*^; CUS-129^*^sv^*) |
| 11DHC (serum) | S | = ^(129^*^sv^*) | ↓ ^(129^*^sv^*) | ↓ (*^Alox5-/-^*^; CUS-129^*^sv^*) |
| 11DHC (F.C. and cerebellum) | S | ↓ ^(129^*^sv^*) | ↓ ^(129^*^sv^*) | = (*^Alox5-/-^*^; CUS-129^*^sv^*) |
| GR (spleen) | S | = ^(129^*^sv^*) | = ^(129^*^sv^*) | = (*^Alox5-/-^*^; CUS-129^*^sv^*) |
| GR (hipoccampus) | S | ↓ ^(129^*^sv^*) | Trend to ↓ ^(129^*^sv^*) (p=0.08) | = (*^Alox5-/-^*) |
| Caspase-1 (adrenal and spleen) | S | ↑ ^(129^*^sv^*) | ↑ ^(129^*^sv^*) | = (*^Alox5-/-^*) ; ↓ ^(CUS-129^*^sv^*) |
|  | | | | |

The arrows indicate an increase or decrease in comparison with the group in the parentheses, and the sign “=” indicates no change. F.C. =

Frontal Cortex; S = Standard; N.D. = Not Determined.
